# KLF5 Is Activated by Gene Amplification in Gastric Cancer and Is Essential for Gastric Cell Proliferation

**DOI:** 10.3390/cells10051002

**Published:** 2021-04-24

**Authors:** Wei Chen, Jian Zhang, Huafeng Fu, Xun Hou, Qiao Su, Yulong He, Dongjie Yang

**Affiliations:** 1 Center for Gastrointestinal Surgery, The First Affiliated Hospital, Sun Yat-Sen University, Guangzhou 510080, China; chenw296@mail.sysu.edu.cn (W.C.); dr.jian.zhang.phd@gmail.com (J.Z.); 18819481474@163.com (H.F.); kris.hou@foxmail.com (X.H.); 2 Department of Pathology, The Seventh Affiliated Hospital, Sun Yat-Sen University, Shenzhen 518107, China; 3 Laboratory of Surgical Department, The First Affiliated Hospital, Sun Yat-Sen University, Guangzhou 510080, China; 4 Animal Experiment Center, The First Affiliated Hospital, Sun Yat-Sen University, Guangzhou 510080, China; 5 Digestive Medicine Center, The Seventh Affiliated Hospital, Sun Yat-Sen University, Shenzhen 518107, China

**Keywords:** KLF5, gastric cancer, copy number variation, TCGA

## Abstract

Gastric cancer is the third leading cause of cancer death worldwide. In this study, we tried to clarify the function of *KLF5* in gastric cancer. Copy number variation (CNV) and the expression of *KLF5* were interrogated in public datasets. The clinical significance of *KLF5* amplification and gene expression in gastric cancer were evaluated. The function of *KLF5* in cell proliferation was studied in gastric cancer cell lines and organoids. We found that *KLF5* amplification mainly occurred in the chromosome instable tumors (CIN) and was significantly associated with *TP53* mutation. In addition, higher *KLF5* expression correlated with more locally invasive gastric cancer and higher T stage. Next, a *KLF5* gene expression signature was curated. The genes in the signature were involved in cell development, cell cycle regulation, cell death, suggesting potential roles played by *KLF5*. Functional studies using siRNAs revealed that *KLF5* was essential for the proliferation of gastric cancer cells. Finally, using gastric organoid models, we revealed that the proliferation of organoids was significantly inhibited after the down regulation of *KLF5*. Our study revealed that *KLF5* was amplified and over-expressed in gastric cancer, and it may play an oncogene-like role in gastric cancer by supporting cell proliferation.

## 1. Introduction

Gastric cancer is one of the leading causes of death related to cancer in the world. It is estimated that about one million gastric cancers are diagnosed each year globally [1]. The treatment of gastric cancer, especially for patients with late stage disease is still largely ineffective. A more thorough understanding of the molecular basis of gastric cancer is mandated for developing new therapeutic drugs for gastric cancer.

Gene amplification is a key mechanism applied by cancer to activate oncogenes. Studies revealed that various genes are amplified in gastric cancer, including growth factor receptor family members such as *HER2*, *EGFR*, and *FGFR2* [2]. These amplified genes are potentially druggable for targeted therapy. Drugs such as trastuzumab that targeting *HER2* had been developed and showed good effects in selected gastric cancer patients with *HER2* amplification [3]. The success of anti-HER2 treatment inspires researchers to search for new druggable targets in gastric cancer. In one study using high resolution single nucleotide polymorphism arrays, Deng et al. conducted a comprehensive survey of whole genomic copy number alterations in gastric cancer. Besides well-known copy number variations listed above, they identified some new locus including *KLF5* (22/233, 9.4%) [4]. Similarly, *KLF5* amplification was also reported by another study in gastric cancer [5]. These data indicated *KLF5* may play a role in gastric cancer.

*KLF5* is a member of the Kruppel-like transcription factor family. Earlier studies found *KLF5* was expressed in dividing cells located at the bottom of the intestinal crypt but was absent in the differentiated cells, suggesting a pro-proliferative role of *KLF5* [6]. This is reflected by the name of *KLF5*, i.e., intestinal-enriched Kruppel-like factor. Following studies using various disease models showed *KLF5* was associated with cell proliferation, metastasis and cell apoptosis [7,8]. The study of *KLF5* in gastric cancer is still limited. Two studies investigated the expression of *KLF5* in gastric cancer samples, but arrived at different conclusions about the prognostic value of *KLF5* [9,10]. The discrepancy highlights the need for more studies of *KLF5* in independent cohort of gastric cancer patients.

The Cancer Genome Atlas (TCGA) program has generated large quantities of genome data in all major cancer types, including gastric cancer [11]. Using TCGA data and other public data sets, we studied the copy number variation (CNV) and gene expression of *KLF5* in gastric cancer. We also curated a consensus list of genes that were possibly regulated by *KLF5*. We further validated *KLF5* expression in an independent cohort of gastric cancer samples from our own institution. The function of *KLF5* was studied using gastric cancer cell lines as well. Our data supported an oncogene-like role played by *KLF5* in gastric cancer.

## 2. Materials and Methods

### 2.1. Patients and Samples

Paired gastric cancer and adjacent normal gastric mucosa samples were randomly selected from our tissue bank and subjected to real-time PCR assay. All participants provided their informed consent before samples were collected for biobanking. The study was conducted in accordance with the Declaration of Helsinki, and the protocol was approved by the Ethics Committee of The First Affiliated Hospital, Sun Yat-sen University (Project identification code: 2012-233). Of 83 pairs of tissues tested, 74 passed quality control and were included in the following analysis. All patients had sporadic gastric cancer and were diagnosed between 2004 and 2009. The age of the patients ranged from 25 to 84 years. All patients have no prior chemotherapy or radiotherapy and underwent radical gastrectomy with D2 lymph node dissection or more. Tissue samples were collected immediately after the removal of the tumor bulk during surgery and stored in ultra-low refrigerator. The diagnosis of all tumors was confirmed by routine histopathological examination. Tumors were staged according to the AJCC TNM staging system (7th edition).

### 2.2. Public Datasets

TCGA (The Cancer Genome Atlas) datasets are either accessed through cBioPortal [11] or downloaded from TCGA data portal directly (https://tcga-data.nci.nih.gov/tcga/ (accessed on 1 January 2021). Microarray data were obtained from the Gene Expression Omnibus (GEO, http://www.ncbi.nlm.nih.gov/geo/ (accessed on 1 January 2021)). Four GEO datasets were analyzed to derivate a consensus *KLF5* signature in this study. All four datasets are whole transcriptome profiling studies comparing *Klf5* knockout versus wild-type mouse tissues. The accession ID of these datasets are GSE65020 [12], GSE58719 [13], GSE39624, GSE27014 [14].

The acronyms for the cancer types are as follows: adrenocortical carcinoma(ACC), bladder urothelial carcinoma (BLCA), breast invasive carcinoma (BRCA), cervical squamous cell carcinoma and endocervical adenocarcinoma (CESC), colorectal adenocarcinoma (COAD), esophageal carcinoma (ESCA), glioblastoma multiforme (GBM), head and neck squamous cell carcinoma (HNSC), kidney chromophobe (KICH), kidney renal clear cell carcinoma (KIRC), kidney renal papillary cell carcinoma (KIRP), acute myeloid leukemia (LAML), brain lower grade glioma (LGG), liver hepatocellular carcinoma (LIHC), lung adenocarcinoma (LUAD), lung squamous cell carcinoma (LUSC), Mesothelioma (MESO), ovarian serous cystadenocarcinoma (OV), pancreatic adenocarcinoma (PAAD), pheochromocytoma and paraganglioma (PCPG), prostate adenocarcinoma (PRAD), Rectum adenocarcinoma (READ), sarcoma (SARC), skin cutaneous melanoma (SKCM), stomach adenocarcinoma (STAD), papillary thyroid carcinoma (THCA), uterine corpus endometrial carcinoma (UCEC), and uterine carcinosarcoma (UCS).

### 2.3. RNA Preparation and Real-Time PCR

Tissue RNA was extracted using Trizol reagent (Invitrogen, Carlsbad, CA, USA). One microgram of total RNA was reverse transcripted into cDNA. Real-time PCR was performed using SYBR Premix Ex Taq kit (Takara, Japan) using ABI 7900HT real-time PCR (Life Technologies, Carlsbad, CA, USA). The PCR primers are as following: *KLF5*, 5′-CCTCCATCCTATGCTGCTAC-3′ (forward), 5′-TTCTCCAAATCGGGGTTACT-3′ (reverse); *GAPDH*: 5′-GCACCGTCAAGGCTGAGAAC-3′ (forward), 5′-TGGTGAAGACGCCAGTGGA-3′ (reverse); *CDKN1C*, 5′-AGATCAGCGCCTGAGAAGTCGT-3′ (forward), 5′-CTCGGGGCTCTTTGGGCTCT-3′ (reverse); *RASSF7*, 5′-CAGCAGAGCGAGCCTTGCAGGCTCA-3′ (forward), 5′-CTGAGTGCCAGGAGGGCCCCTGTCA-3′ (reverse); *GPRC5A*, 5′-GCTGCTCACAAAGCAACGAA-3′ (forward), 5′-ATAGAGCGTGTCCCCTGTCT-3′ (reverse); *MPZL2*, 5′-GCTTTTCCAGTTGTGACCCG-3′ (forward), 5′-CTCCAGAGGGGTGTTGCTTG-3′ (reverse).

### 2.4. siRNA Transfection and Western Blot

The human gastric cancer cell line SGC7901 was purchased from the Type Culture Collection of the Chinese Academy of Sciences (Shanghai, China). Cells were maintained in RPMI1640 (Hyclone, MA, USA) containing 10% fetal bovine serum and cultured in a humidified atmosphere of 5% CO2 at 37 °C. The small interfering RNA against human KLF5 was synthesized by RiboBio (Guangzhou, China). The targeted sequence was: *KLF5*: 5′-AGCTCACCTGAGGACTCACAC-3′ (siKLF5-1) [15]; 5′-CGATTACCCTGGTTGCACA-3′ (siKLF5-2). Luciferase control (CON-si): 5′-CTTACGCTGAGTACTTCGA-3′. siRNAs were delivered using Lipofectamin 2000 reagent (Life Technologies, MD, USA) according to standard protocol. Western blot was probed with antibodies against GAPDH (bsm-0978M, Bioss, Beijing, China) or KLF5 (ab24331, Abcam, Cambridge, UK).

### 2.5. Cell Proliferation Assay, Clone Formation Assay

24 h after siRNA transfection, cells were trypsinized and seeded in a 96-well plate at a density of 1500 cells per well. Then cell viability was analyzed by MTT assay after three days. For clone formation assay, cells were seeded in a 6-well plate at 1000 cells per well. Culture media was changed every 3 days for 2 weeks. Then cell clones were stained with 0.1% crystal violet. The protocol for the establishment and maintenance of gastric organoids was described in our previous study [16]. Organoids were digested into single cells and counted with a hemocytometer. A total of 10,000 cells were used in the transfection of each siRNA. Then cells were spin down, mixed with Matrigel, and seeded into 5 wells of a 96-well plate for each siRNA group. The culture medium was changed every two days. Organoids were photographed at day 7 post-transfection. The experiment was repeated 3 times, and one typical result was shown.

### 2.6. Statistical Analysis

All statistical analyses in this study were performed using the R statistical software (https://www.r-project.org (accessed on 1 January 2021)). For real-time PCR analysis, gene expression levels were represented by CT (threshold cycle) value. Gene expression was normalized using ΔCT (dCT) by subtracting the CT value of *GAPDH* from that of the target gene. ΔΔCT was calculated by subtracting ΔCT of the control sample from that of the experiment sample. Fold changes were calculated using the formula: 2^−ΔΔCT^. Group means were compared using paired one-sample t-test when analyzing gene expression in tissue samples. The association of expression levels between different genes was assessed with Pearson correlation coefficient. For TCGA RNA sequencing data, the normalized RPKM data was log2 transformed. For GEO datasets, the series matrix files were downloaded. Data was quantile normalized. Differential expression tests were performed using limma package from R. Kaplan–Meier method was used to estimate survival curves. Cox proportional hazards regression was used for multivariate survival analysis. *P*-values were two-sided, and *p* ≤ 0.05 was considered statistically significant. All cell experiments had been repeated at least 3 times.

## 3. Results

### 3.1. KLF5 Showed Both Gene Amplifications and Deletions in Pan-Cancer

**Figure 1:**
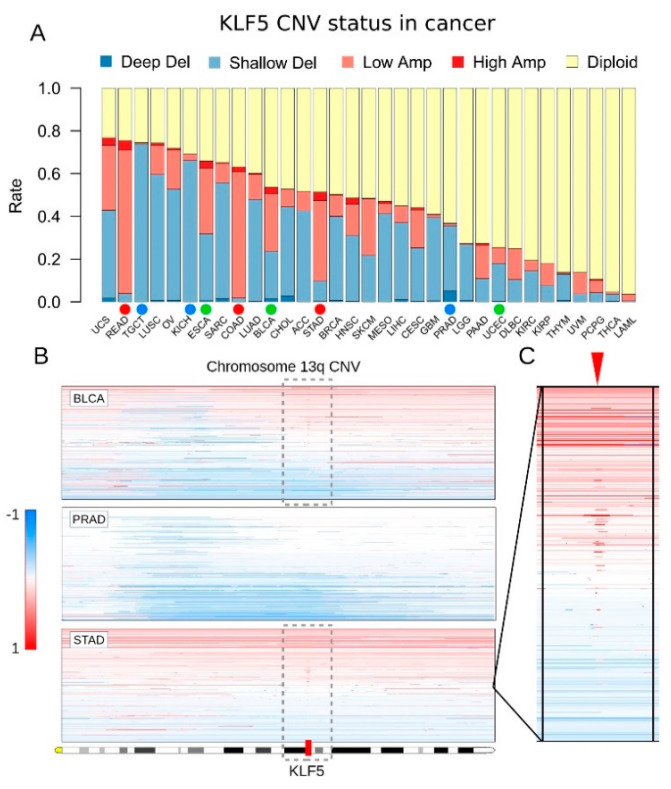
*KLF5* copy number variations (CNV) in 33 cancer types. (**A**) Barplot showing the proportions of *KLF5* CNV across 33 tumor types. The full name of each tumor type is given in the Public datasets section from the Materials and Methods. The dots below the bars indicate different types of CNV status of *KLF5*. The red dot indicates *KLF5* is mainly amplified, the blue dot indicates *KLF5* is mainly deleted, whereas the green dot indicates similar amplification and deletion rates of *KLF5* in that tumor type. (**B**) Heatmap showing chromosome 13q copy number changes in three cancer types. In gastric cancer (STAD) and a subset of bladder cancer (BLCA), whole chromosome arm 13q duplication, as well as regional amplification, accounted for gaining of copy number in *KLF5*. In prostate cancer (PRAD) or other parts of bladder cancer, long segment deletion of chromosome 13q accounted for the loss of copy number in *KLF5*. (**C**) The room-in view of chromosome 13q that carries *KLF5* amplification in gastric cancer. In the heatmap, red stands for amplification, blue stands for deletion, and white stands for the diploid gene.

Copy number variation (CNV) of *KLF5* is prevalent in different tumor types, according to a previous study [17]. We first studied the CNV of *KLF5* across a large series of cancer samples from TCGA dataset (Figure 1A). In pan-cancer of 33 tumor types, the *KLF5* gene showed both amplifications and deletions in different tumor types. The rates of *KLF5* variation ranged from 3.6% (acute myeloid leukemia, LAML) to 76.8% (uterine carcinosarcoma, UCS). Specifically, *KLF5* was prone to be deleted in prostate cancer (PRAD) but amplified in gastric cancer (STAD), colorectal cancer (COAD, READ), and head and neck cancer (HNSC). Interestingly, both amplification and deletion of *KLF5* could be found at a similar rate in some cancer types, such as uterine carcinosarcoma (UCS) and bladder cancer (BLCA). As for gastric cancer, among 441 primary tumor samples studied, 182 tumors had *KLF5* amplification (41.4%), whereas 43 tumors had *KLF5* deletion (9.8%) (Figure 1A). In addition, the CNV of *KLF5* was mainly comprised of shallow deletions and low amplifications rather than deep deletions or high-level amplifications (Figure 1A).

We then manually checked *KLF5* CNV across different cancer types using a genome browser. We found that the *KLF5* locus was affected by both arm-level chromosome gains and losses, as well as local amplification (Figure 1B,C). In gastric cancer and a subset of bladder cancer, there were duplications of chromosome 13q as well as regional amplification of a region close to *KLF5* (Figure 1B,C). In prostate cancer or another subset of bladder cancer, long segment deletion of chromosome 13q accounted for the loss of *KLF5* (Figure 1B). Recent reports demonstrated that focal amplification close to the *KLF5* locus acted as an enhancer and can activate *KLF5* expression [5,17].

### 3.2. KLF5 Copy Number Showed a Dose-Response Effect on Its mRNA Expression

**Figure 2:**
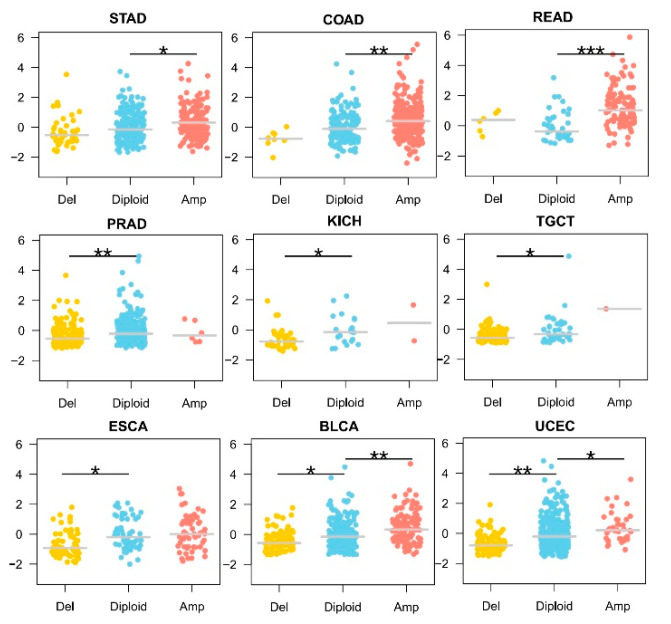
Relationship between *KLF5* copy number and gene expression in different tumor types. In gastrointestinal tumors with predominantly *KLF5* amplification, including gastric cancer (STAD), colon cancer (COAD), and rectum cancer (READ), an increase in *KLF5* transcripts could be observed (top 3 figures). For prostate cancer (PRAD), kidney chromophobe (KICH), and testicular germ cell tumors (TGCT), *KLF5* deletion is predominant, and a decrease in *KLF5* transcription could be observed (middle 3 figures). For other tumor types such as esophageal carcinoma (ESCA), bladder urothelial carcinoma (BLCA) and uterine corpus endometrial carcinoma (UCEC), which have comparable rates of *KLF5* amplification and deletion, *KLF5* copy number changes and gene expression shows a dose-response relationship (bottom 3 figures). * 0.05 < *p* < 1 × 10^−4^, ** 1 × 10^−4^ < *p* < 1 × 10^−6^, *** *p* < 1 × 10^−6^.

We then asked whether CNV of *KLF5* had any functional effects on its mRNA expression. In gastrointestinal tumors with predominantly *KLF5* amplification, including gastric cancer (STAD), colon cancer (COAD), and rectum cancer (READ), an increase in *KLF5* transcripts could be observed (Figure 2, top 3). For prostate cancer (PRAD), kidney chromophobe (KICH), and testicular germ cell tumors (TGCT), *KLF5* deletion was predominant, and a decrease in *KLF5* transcription could be observed (Figure 2, middle 3). For other tumor types such as esophageal carcinoma (ESCA), bladder urothelial carcinoma (BLCA) and uterine corpus endometrial carcinoma (UCEC), which had comparable rates of *KLF5* amplification and deletion, *KLF5* expression changed in accordance to its copy number (Figure 2, bottom 3). Thus, the CNV of *KLF5* seemed to affect *KLF5* expression. In addition, the copy number change in *KLF5* and its mRNA expression appeared to have a dose-response relationship.

### 3.3. KLF5 Correlated with More Locally Invasive Gastric Cancer

**Figure 3:**
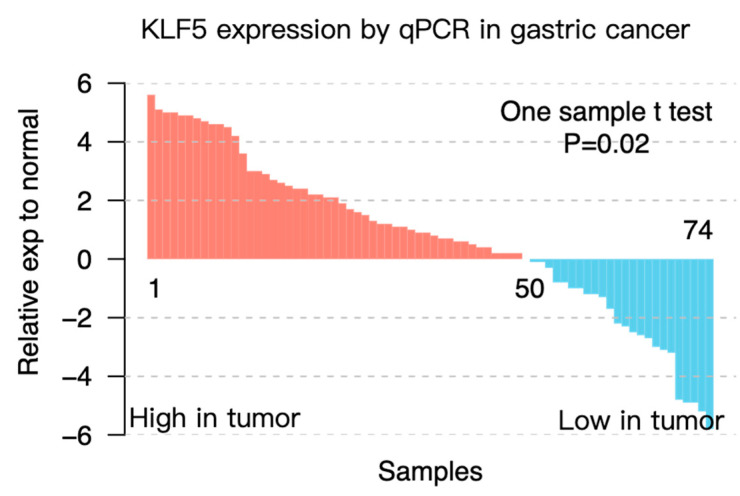
Expression of *KLF5* in an independent cohort of gastric cancer samples. *KLF5* expression was evaluated by real-time PCR in 74 pairs of gastric cancer and adjacent normal gastric tissues. Gastric cancer tissues showed higher *KLF5* expression compared with adjacent normal control tissues (one-sample *t*-test: *p* = 0.02).

To confirm the above findings, we tested the mRNA expression of *KLF5* in 74 pairs of gastric cancer tissues and their normal counterparts using real-time PCR. Patients were divided into *KLF5* high or low groups by comparing *KLF5* expression in tumors with corresponding adjacent normal tissues. A total of 50 out of 74 tumors showed higher expression of *KLF5* (*t*-test: *p* = 0.02) (Figure 3). We found that higher expression of *KLF5* correlated with larger tumor size (*p* = 0.008) and later tumor (T) stage (*p* = 0.029) (Table 1). In addition, tumors with higher *KLF5* expression also tended to invade the serosal covering of the stomach (*p* = 0.054), which was also a sign of invasiveness (Table 1). These data demonstrated that higher KLF5 expression was positively correlated with the tumor burden in gastric cancer.

### 3.4. KLF5 Was Associated with the Molecular Features of Gastric Cancer

**Figure 4:**
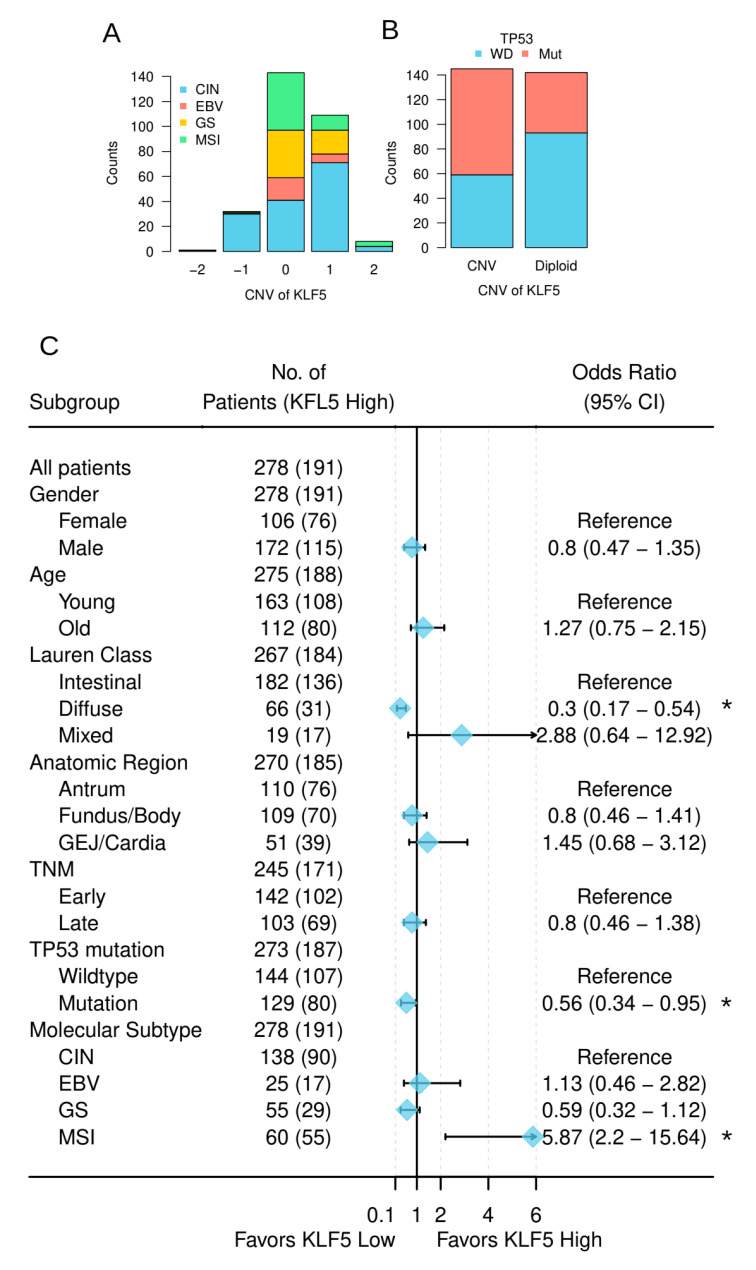
Relationship between *KLF5* and characteristics of gastric cancer from TCGA cohort. (**A**) Relationship between the CNV of *KLF5* and the molecular subtypes of gastric cancer (Supplementary Table S1); (**B**) relationship between *KLF5* copy number and *TP53* mutation status. WD stands for wild-type, Mut stands for mutated (Supplementary Table S2); (**C**) association between *KLF5* expression and the clinicopathological features of the patients from TCGA cohort. * *p* < 0.05.

The TCGA gastric cancer study proposed a four-category molecular classification of gastric cancer, which is considered to be the most comprehensive and clinically relevant. Our correlation analysis indicated that tumors carrying *KLF5* CNVs were mainly from the CIN (chromosome instability) subtype (chi-square test: *p* = 1.6 × 10^−12^) (Figure 4A). This result suggested CNV of *KLF5* was related to the overall chromosomal instability of gastric cancer. As *TP53* is well-known for its role in maintaining chromosomal stability, we next assessed correlation between *KLF5* CNV and *TP53* mutation. As shown in Figure 4B, *KLF5* CNV was significantly associated with *TP53* mutation (chi-square test: *p* = 4.3 × 10^−5^).

**Figure 5:**
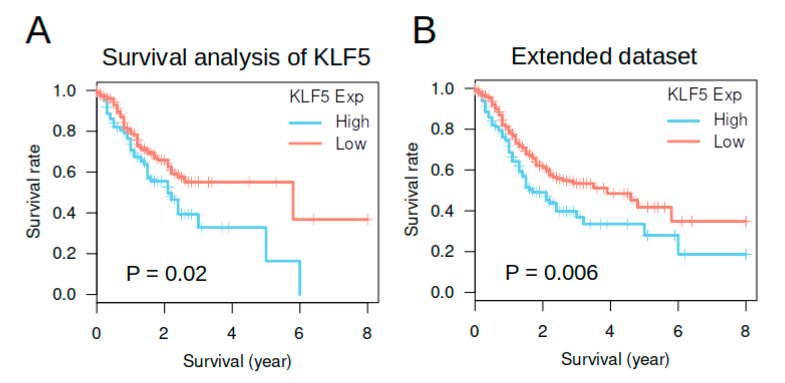
Survival analysis of *KLF5* expression in gastric cancer from TCGA dataset. (**A**,**B**) showed the Kaplan–Meier plots of survival analysis. *P*-values in the figures were calculated by log-rank tests. (**A**) came from a smaller dataset containing 278 patients with molecular classification, whereas (**B**) came from an extended dataset containing 414 patients with good follow-up data.

Correlation analysis between *KLF5* expression and the clinicopathological characteristics of the patients revealed similar results in TCGA datasets as in our own cohort (Figure 5). In addition, Lauren classification and molecular features of the tumors also correlated with *KLF5* expression in TCGA datasets (Figure 4C). Specifically, *KLF5* high expression tumors were enriched in MSI (microsatellite instable) subtype (odds ratio = 5.9, *p* = 0.0001). *KLF5* high expression tumors were less represented in the diffused type of gastric cancer (odds ratio = 0.3, *p* = 4.0 × 10^−5^) and *TP53* mutated samples (odds ratio = 0.56, *p* = 0.03) (Figure 5). In multivariate logistic regression analysis, the diffused type of Lauren classification, *TP53* mutation, and the MSI subtype of molecular classification were independent predictors of *KLF5* expression (Supplementary Table S3).

### 3.5. KLF5 Expression Was Not an Independent Prognosis Factor in TCGA Gastric Cancer Cohort

We then performed survival analysis using TCGA dataset to see whether *KLF5* had prognostic value in gastric cancer. Tumors were dichotomized into two groups using the median of *KLF5* expression in normal gastric tissue as a cutoff. In a smaller dataset (278 patients) containing only patients with molecular classification information, Kaplan–Meier analysis indicated higher *KLF5* expression was related to better survival (Log-rank tests: *p* = 0.02) (Figure 5A). In an extended dataset containing 414 patients, higher *KLF5* expression was also related to better survival (log-rank tests: *p* = 0.006) (Figure 5B). These data suggested *KLF5* may be a good prognostic factor for gastric cancer in TCGA dataset.

As MSI status and the diffused pathological class were well-known prognostic factors for gastric cancer, we thus performed multivariate survival analysis including these factors and TNM stage to assess the prognostic value of *KLF5* expression. In univariate analysis, *KLF5* expression, TNM stage and Lauren classification were significant prognostic factors for gastric cancer. When including all variables into the model, *KLF5* was no longer a statistically significant prognostic factor (*p* = 0.22) (Table 2). On the other hand, TNM stage and the mixed type of Lauren classification remained as the significant prognostic factor. These data suggested *KLF5* expression was not an independent prognostic factor for gastric cancer.

### 3.6. Derivation of an Aonsensus Gene Expression Signature Associated with KLF5

**Figure 6:**
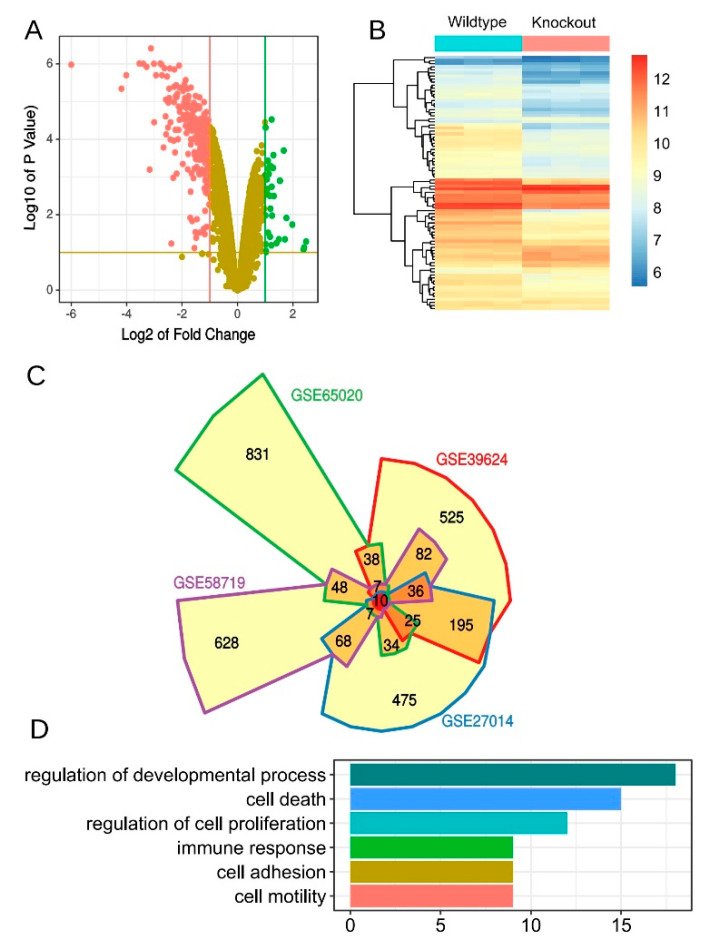
A consensus gene signature that is related to *Klf5* knockdown. (**A**) Volcano plot showing genes with significant fold changes between *Klf5* knockout versus *Klf5* wild-type mouse samples in GSE39624 dataset; (**B**) heatmap showing the expression of an 84-gene signature in GSE39624 dataset; (**C**) top differentially expression genes from four independent datasets were compared and visualized by the Venn diagram; (**D**) gene ontology analysis of the 84-gene signature. Bars represented the number of genes mapped into each gene set.

Although acting as a transcription factor, the consensus target genes of *KLF5* is still unknown yet. We tried to curate a *KLF5* regulated gene list by polling different studies of *Klf5* transgenic models. A thorough search in the GEO database returned five studies with similar design (Supplementary Table S4) [12,13,14]. All five studies profiled whole transcriptome of *Klf5* knockout transgenic mice. Four studies applied microarray technology, whereas the fifth one used high throughput sequencing and thus was not used for curation of the signature. Differential gene expression (DE) analysis was performed for each dataset. We observed high skew toward gene down regulation when *Klf5* was knockout in all four studies. This was exemplified by the volcano plot and the heatmap showing differentially expressed genes comparing *Klf5* knockout versus wild-type control samples (Figure 6A,B). Actually, 72–98% of top 100 DE genes were downregulated in four datasets. This result suggested knockout of *Klf5* had a significant effect on the overall transcriptome of tissues, reiterating its important role in stem cell and development.

The top 1000 differentially expressed genes ranked by adjusted *p*-values from four studies were polled to derive a consensus gene list (Figure 6C, Supplementary Table S5). A total of 84 genes that were present in three or more DE lists were considered as most commonly affected genes after *Klf5* was knockout. Of the 84 genes, *Cdkn1c* (cyclin-dependent kinase inhibitor 1C) was among the few genes that were upregulated after the knockout of *Klf5* (Supplementary Table S5). *Cdkn1c* is a strong inhibitor of several cyclin/Cdk complexes and a negative regulator of cell proliferation [18]. *Rassf7* (Ras-association domain family member 7) negatively regulates stress-induced JNK activation and apoptosis by promoting MAP2K7 phosphorylation [19,20,21]. *Gprc5a* is a member of the type 3 G protein-coupling receptor family and may play a role in embryonic development and epithelial cell differentiation [22,23]. *Mpzl2* (myelin protein zero-like 2) is highly homologous to the myelin protein zero and is expressed in the thymus and in several epithelial structures early in embryogenesis [24]. Gene ontology (GO) analysis revealed several highly enriched GO terms, including regulation of development, cell death, cell proliferation, et al. (Figure 6D).

**Figure 7:**
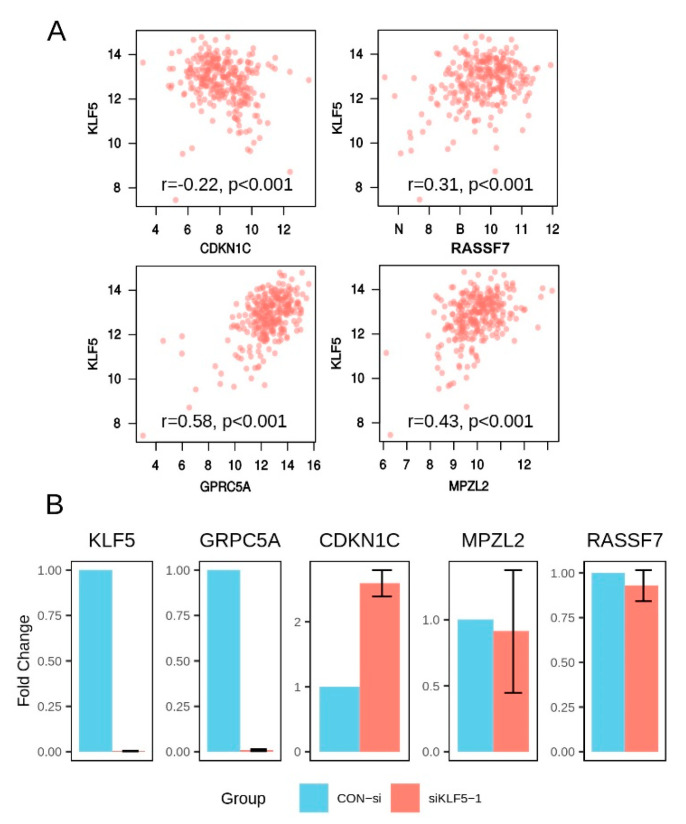
Screening and validation of potentially *KLF5* regulated genes. (**A**) Scatter plots showing the correlation between the expression of *KLF5* and selected genes in TCGA dataset. (**B**) qualitative PCR analysis of gene expression after transient downregulation of *KLF5* using siRNA in SGC7901. *KLF5*, *GRPC5A*, and *CDKN1C* showed statistically different expressions between siKLF5-1 and control treatment groups (*T*-test: *p* < 0.05 for all three genes). The assay was repeated three times and one of the typical results was shown here.

Next, we asked whether these correlations were also valid in human gastric cancer samples. We first mapped the top 20 mouse genes of the 84-gene signature into their human orthologs and obtained 18 genes. Then the relation between the expression of *KLF5* and these genes was assessed in human gastric cancer tissues. We found that most of correlations discovered through transgenic mice studies were also valid in gastric cancer tissues (Table 3). For example, expression of *CDKN1C*, *RASSF7*, *GPRC5A*, and *MPZL2* were all significantly related to *KLF5* in gastric cancer tissue (Figure 7A and Table 3). Five genes, e.g., *DGAT2*, *JAKMIP1*, *KRT7*, *PGLYRP1*, and *TINAGL1*, showed very low correlation coefficient and/or insignificant p-values, suggesting they might not be regulated by *KLF5* in human gastric cancer (Table 3). To validated these findings, we transiently down regulated *KLF5* expression using siRNA and then assessed the expression of four selected genes, including *CDKN1C*, *RASSF7*, *GPRC5A*, and *MPZL2*. After the silencing of *KLF5*, *GPRC5A* expression was significantly downregulated (Figure 7B). On the other hand, *CDKN1C* showed increased expression by about two folds (Figure 7B). However, no significant change was observed in the expression *RASSF7* or *MPZL2* (Figure 7B). Using another *KLF5* siRNA, the similar result was also observed with upregulation of *CDKN1C* and downregulation of *GPRC5A* (Supplementary Figure S1).

### 3.7. KLF5 Regulated the Proliferation of Gastric Cancer CSells

**Figure 8:**
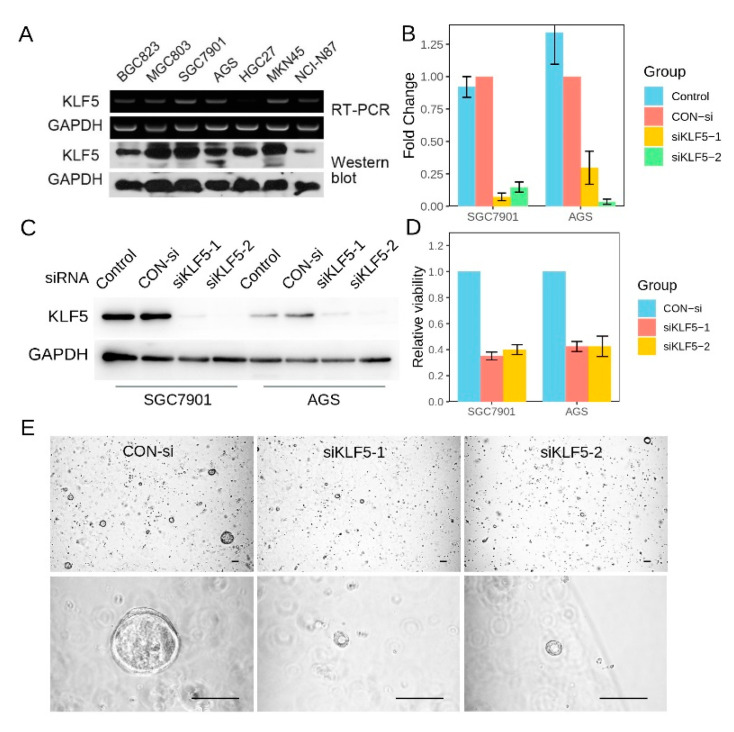
*KLF5* was essential for the proliferation of gastric cancer cells. (**A**) *KLF5* mRNA (RT-PCR) and protein (Western blot) expression in seven gastric cancer cell lines; (**B**) RT-PCR showing that *KLF5* specific siRNA could downregulate *KLF5* mRNA effectively in two gastric cancer cell lines; (**C**) Western blot confirmation of *KLF5* downregulation in gastric cancer cell lines; (**D**) MTT assay showing that cell proliferation was significantly compromised after *KLF5* was downregulated by siRNA; (**E**) the proliferation of gastric organoids was significantly reduced when *KLF5* was downregulated by siRNA, as indicated by lower organoid formation efficiency and smaller organoid size. Abbreviations: RT-PCR, real-time PCR; CON-si, control siRNA. The scale bar indicated 100 μm.

We then tested *KLF5* expression in a series of gastric cancer cell lines using RT-PCR and Western blot. We found that *KLF5* expression varied among gastric cancer cell lines (Figure 8A). SGC7901, MGC803, and MKN45 exhibited high *KLF5* expression, whereas AGS and HGC27 exhibited median *KLF5* expression. In the starvation assay, we observed that *KLF5* expression can be induced by fetal bovine serum (FBS) treatment, which suggested that *KLF5* was responsive to mitogenic factors (Supplementary Figure S1). We then explored the function of *KLF5* in gastric cancer cell lines using *KLF5* siRNAs. After cells were transfected with KLF5-siRNA for 72 h, the expression of *KLF5* was down regulated at both transcripts and protein levels (Figure 8B,C). At the same time, the viability of cells decreased significantly compared with that of the control groups in both cell lines tested (Figure 8D). In addition, when *KLF5* expression was silenced, SGC7901 showed less clone formation ability than the cells treated with control siRNA (Supplementary Figure S1). To confirm the role of *KLF5* in regulating cell proliferation, we also silenced *KLF5* expression in a normal gastric organoid. The proliferation of gastric organoid was significantly reduced when *KLF5* was downregulated by siRNA, as indicated by lower organoid formation efficiency and smaller organoid size (Figure 8E). This evidence demonstrated that *KLF5* was essential for the proliferation of gastric cancer cells.

## 4. Discussion

The roles played by *KLF5* in cancer have been controversial. Most studies label *KLF5* as an oncogene. For example, *KLF5* has been shown to transform non-malignant NIH3T3 cells [25]. It also promotes the growth of breast cancer [15], bladder cancer [26] and is essential for the anchorage-independent growth of lung cancer cells [27]. In addition, the expression of *KLF5* in breast cancer correlates with both poorer disease-free survival and overall survival [28]. However, a few other studies reveal a growth inhibitory role of *KLF5*. For example, in a different breast cancer cell line, *KLF5* was shown to have growth inhibitory functions [29]. In addition, *KLF5* was inactivated by the hemizygous deletion in prostate cancer, and re-expression of *KLF5* inhibits cell growth in vitro [30]. To reconcile these different observations, some researchers suggested the role of *KLF5* in cancer may be context-dependent. In other words, *KLF5* may exert pro-cancer or anti-cancer functions depending on the different cancer types studied [31].

Through analyzing the copy number variation data of a large cohort of tumors from 33 different cancer types, we found opposite changes in CNV of *KLF5* occurred in different tumor types. In gastrointestinal cancers, including gastric cancer and colorectal cancer, *KLF5* was predominantly amplified. In prostate cancer, however, *KLF5* was exclusively deleted. In most other tumor types, both *KLF5* amplification and deletion were observed. Our observation was consistent with the results from the previous functional analysis mentioned above. It seems that the dose of *KLF5 copy* number is critical, with too much of *KLF5* (chromosome amplification) or too little of KLF5 (chromosomal deletion) leading to overactivation or downregulation of its function, respectively. Thus, our observations may partially explain the long-standing conflict regarding the functional assertions of *KLF5* in different studies.

Evidence supporting the involvement of *KLF5* in proliferation and tumorigenesis of the digestive tract has been accumulating. In one study using ED-L2/KLF5 transgenic mice, overexpression of *KLF5* in esophageal epithelia induced increased proliferation of cells in the basal layer [32]. In another study, heterozygous *KLF5* knockout (*KLF5*(+/−)) mice showed decreased colonic crypt height when treated with inflammatory stimuli [33]. Both of the above experiments show a critical role of *KLF5* in sustaining cell growth. More importantly, in ApcMin/KRAS V12 double transgenic mice that had a great tendency to develop small intestine tumors, deleting one allele of *Klf5* led to a 92% reduction in tumor burden [34]. This evidence supports the potential role of *KLF5* in the tumorigenesis of the digestive tract.

While the role of *KLF5* in gastrointestinal tract development and the formation of intestinal tumors has been demonstrated extensively, its function in gastric cancer is still unclear. One study using microarray revealed that the *KLF5* gene locus was amplified in around 10% of gastric cancer [4]. In our analysis focusing on both low and high *KLF5* amplifications, we found *KLF5* gene was amplified in 41.4% of gastric cancer tissue from TCGA dataset. The expression of *KLF5* in gastric cancer samples has also been studied by other investigators using immunohistochemistry [9,10]. In one study, *KLF5* was found to be expressed in 45.7% of samples. These patients exhibited an early-stage disease and favorer survival [9]. However, a later study revealed that the nuclear staining of *KLF5* was associated with more advanced cancer and poorer survival [10]. These conflicting results hampered our interpretation of the roles played by *KLF5* in gastric cancer.

In our cohort, *KLF5* mRNA was elevated in 67.6% (50/74) gastric cancer samples. In addition, higher *KLF5* expression correlated with more locally invasive gastric cancer as indicated by bigger tumor size and higher T stage. However, *KLF5* expression in gastric cancer is not a meaningful prognostic factor for the patients. Although validation using TCGA cohort showed higher *KLF5* expression was associated with better survival, it was not an independent prognostic factor when considering confounding factors, including tumor stage and molecular classification. Thus, our analysis suggested the prognostic value of *KLF5* expression is limited. This is probably because *KLF5* expression was correlated with the T stage of tumor and the Lauren classification, which was suggestive of the potential biological roles played by *KLF5*.

As the function of *KLF5* is content-dependent, we need to study *KLF5* in a case-by-case manner in different cancer types. In the two studies investing expression of *KLF5* in gastric cancer tissue, no functional experiment was performed [9,10]. One recent study silenced *KLF5* expression using siRNAs and demonstrated that *KLF5* downregulation inhibited the proliferation of gastric cancer cells [35]. The results of our study using a different gastric cancer cell line confirmed their findings. In addition, we also observed decreased clone formation ability of gastric cancer cells when *KLF5* was silenced. These data, together with the observation that *KLF5* was activated upon FBS treatment, demonstrated *KLF5* was essential for the growth of gastric cancer.

The transcriptional factor *KLF5* is a multi-function gene. It has thus far been linked to many signaling pathways, including WNT, RAS, TGFβ, Hippo, Notch, retinoid acid receptor, and hormone receptors [31]. One major problem is that no consensus genes that are regulated by *KLF5* have been reported. To address this problem, we analyzed four transcriptomic studies with a similar experimental design. A consensus gene list including *Klf5* was derived. Interestingly, many genes in this consensus list were involved in the regulation of development, cell cycle, and cell death, which are important traits of cancer. Among these genes, *Cdkn1c* was most special, as it was the only gene that shows higher expression after *Klf5* knockout. This observation suggests *Klf5* may negatively regulate *Cdkn1c* expression. As *Cdkn1c* is an inhibitor of G1 cyclin/Cdk complexes and a negative regulator of the cell cycle, it is possible that *Klf5* lifts the barrier imposed by *Cdkn1c* on cell cycle, thereby promoting cell proliferation. Apart from *Cdkn1c*, other cancer-related genes, such as *RASSF7* and *GPRC5A* has also been linked with *Klf5* in this study. More experimental studies are warranted to verify whether *KLF5* exerts its growth-promoting effects through regulating those genes in the future.

## 5. Conclusions

In summary, this study revealed that *KLF5* underwent amplification or deletion in different tumor types, which led to up- or downregulation of *KLF5* mRNA expression accordingly. In gastric cancers, *KLF5* was suffered from arm-level as well as focal amplification. *KLF5* amplification was significantly associated with genome-unstable tumors and *TP53* mutation. *KLF5* expression correlated with a more locally invasive disease but was not an independent prognostic factor. Functional assays revealed *KLF5* was essential for the proliferation of gastric cancer. Collectively, these data demonstrated *KLF5* played an oncogene-like role in gastric cancer.

## Figures and Tables

**Table 1:** Clinicopathological characteristics of the patients grouped by KLF5 Expression.

	Total	KLF5 Expression (Low)	KLF5 Expression (High)	*P*
Cases	74	24	50	
Sex				0.296
Male	55 (74.3%)	16 (66.7%)	39 (78.0%)	
Female	19 (25.7%)	8 (33.3%)	11 (22.0%)	
Age (year)				0.909
≤55	24 (32.4%)	8 (33.3%)	16 (32.0%)	
>55	50 (67.6%)	16 (66.7%)	34 (68.0%)	
Length (cm)				0.008 **
≤5	39 (52.7%)	18 (75.0%)	21 (42.0%)	
>5	35 (47.3%)	6 (25.0%)	29 (58.0%)	
Serosa Invasion				0.054
Negative	58 (78.4%)	22 (91.7%)	36 (72.0%)	
Positive	16 (21.6%)	2 (8.3%)	14 (28.0%)	
Primary Tumor (T)				0.029 *
T1/T2	19 (25.7%)	10 (41.7%)	9 (18.0%)	
T3/T4	55 (74.3%)	14 (58.3%)	41 (82.0%)	
Regional Lymph Nodes (N)				0.619
N0/N1	37 (50.0%)	13 (54.2%)	24 (48.0%)	
N2/N3	37 (50.0%)	11 (45.8%)	26 (52.0%)	
Distant Metastasis (M)				0.329
M0	60 (81.1%)	21 (87.5%)	39 (78.0%)	
M1	14 (18.9%)	3 (12.5%)	11 (22.0%)	
TNM				0.189
Ⅰ/Ⅱ	32 (43.2%)	13 (54.2%)	19 (38.0%)	
Ⅲ/Ⅳ	42 (56.8%)	11 (45.8%)	31 (62.0%)	
Histological Grade (G)				0.762
Well/Moderate	29 (39.2%)	10 (41.7%)	19 (38.0%)	
Poor/Undifferentiated	45 (60.8%)	14 (58.3%)	31 (62.0%)	

* *p* < 0.05, ** *p* < 0.01 (chi-square test). The T stage refers to the extent of the primary tumor; the N stage refers to the number of lymph nodes with metastatic cancer; the M stage refers to distant metastasis. Tumors were staged according to the AJCC TNM staging system (7th edition).

**Table 2:** Cox proportional hazards regression model analysis for overall survival.

	Univariate		Multivariate	
	HR (95% CI)	*P*	HR (95% CI)	*P*
KLF5 expression (high vs. low)	0.62 (0.41, 0.94)	0.02 *	0.72 (0.43, 1.22)	0.22
TNM stage (late vs. early)	2.24 (1.44, 3.5)	0.0004 ***	1.8 (1.12, 2.89)	0.02 *
Molecular subtype (EBV vs. CIN)	0.85 (0.4, 1.8)	0.67	0.91 (0.38, 2.14)	0.82
Molecular subtype (GS vs. CIN)	1.11 (0.68, 1.83)	0.67	0.87 (0.45, 1.69)	0.69
Molecular subtype (MSI vs. CIN)	0.74 (0.43, 1.27)	0.27	0.69 (0.34, 1.4)	0.3
Lauren (Diffused vs. Intestinal)	1.61 (1.02, 2.55)	0.04 *	1.29 (0.68, 2.47)	0.44
Lauren (Mixed vs. Intestinal)	2.5 (1.33, 4.7)	0.004 **	2.37 (1.14, 4.92)	0.02 *

Abbreviation: HR, hazards ratio; CI, confidence interval. * *p* < 0.05, ** *p* < 0.01, *** *p* < 0.001.

**Table 3:** Pearson correlation between expression of KLF5 and other genes in TCGA gastric cancer study.

Gene	Correlation	*p*-Value
AIM1L	0.41	1.57 × 10^−12^
CDKN1C	−0.22	1.64 × 10^−4^
CMBL	0.17	5.84 × 10^−3^
DGAT2	0.10	1.02 × 10^−1^
GPRC5A	0.58	3.02 × 10^−26^
GPX2	0.20	1.05 × 10^−3^
JAKMIP1	−0.10	9.61 × 10^−2^
KRT7	0.11	7.44 × 10^−2^
MPZL2	0.43	3.80 × 10^−14^
PGLYRP1	−0.02	7.85 × 10^−1^
PRR13	0.29	1.28 × 10^−6^
RASSF7	0.31	2.08 × 10^−7^
SH2D4A	0.48	2.27 × 10^−17^
SH3BGRL2	0.42	1.95 × 10^−13^
SLC25A10	0.30	5.58 × 10^−7^
TINAGL1	0.06	2.92 × 10^−1^
TJP3	0.51	2.31 × 10^−19^
TSPAN8	0.44	1.58 × 10^−14^

## Data Availability

The data presented in this study are available on request from the corresponding author.

## Supplementary Materials

The following are available online at https://www.mdpi.com/article/10.3390/cells10051002/s1. Figure S1: The relation between KLF5 and cell proliferation. Table S1: Relationship between the CNV of KLF5 and the molecular subtypes of gastric cancer. Table S2: Relationship between KLF5 copy number and TP53 mutation status. Table S3: Multivariate logistic regression analysis for KLF5 expression in gastric cancer. Table S4: Public datasets analyzing the whole transcriptome of KLF5 knockout mice. Table S5: Top 84 differentially expressed genes comparing wild-type and Klf5 knockout mouse across 4 datasets.
