# Supplementary material for: KLF5 Is Activated by Gene Amplification in Gastric Cancer and Is Essential for Gastric Cell Proliferation
Source: Cells. 2021 Apr 24;10(5):1002. doi: 10.3390/cells10051002 (PMC8152363; doi:10.3390/cells10051002)
Supplement: Supplementary file 1 [file cells-10-01002-s001.zip › cells-1172177-supplementary.pdf]

Supplement data

Supplement tables

Supplement table 1. Relationship between the CNV of KLF5 and the molecular subtypes of gastric cancer. Number in the parenthesis is the percentage of all patients.

Copy	-2	-1	0	1	2
**CIN**	1(0.3)	30(10.2)	41(14)	71(24.2)	4(1.4)
**EBV**	0	1(0.3)	18(6.1)	7(2.4)	0
**GS**	0	0	38(13)	19(6.5)	0
**MSI**	0	1(0.3)	46(15.7)	12(4.1)	4(1.4)

Supplement table 2. Relationship between KLF5 copy number and TP53 mutation status. Number in the parenthesis is the percentage of all patients.

KLF5 Copy	CNV	Diploid
TP53 Wildtype	59(20.5)	93(32.4)
TP53 Mutated	86(30)	49(17.1)

Supplement table 3. Multivariate logistic regression analysis for KLF5 expression in gastric cancer.

	Estimate	SD	OR (95% CI)	z	P (>|z|)
Lauren Class (Diffuse)	-1.11	0.39	0.33(0.15,0.71)	-2.83	0.005**
Lauren Class (Mixed)	1.27	0.79	3.55(0.92,23.5)	1.61	0.11
TP53 mutation (Mutation)	-0.99	0.38	0.37(0.17,0.77)	-2.57	0.01*
Molecular Subtype (EBV)	-0.46	0.59	0.63(0.2,2.06)	-0.79	0.43
Molecular Subtype (GS)	-0.49	0.49	0.61(0.23,1.62)	-0.99	0.32
Molecular Subtype (MSI)	1.98	0.64	7.24(2.37,31.63)	3.10	0.002**

Abbreviation: OR, odds ratio; CI, confidence interval. *P < 0.05, **P < 0.01.

Supplement table 4. Public datasets analyzing the whole transcriptome of KLF5 knockout mice

Author	Model	Platform	Accession	Information	Reference
2017 Azami T	Mouse embryos E3.0	Microarray	GSE65020	embryo	[1]
2014 Xing C	Mouse adults	Microarray	GSE58719	prostate	[2]
2012 Bell SM	Mouse embryos E14.5	Microarray	GSE39624	intestine	
2011 Bell SM	Mouse embryos E14.5	Microarray	GSE27014	bladder	[3]
2016 Noah F	Mouse adults	RNAseq	GSE79758	intestine	

Supplement table 5. Top 84 differentially expressed genes comparing wildtype and Klf5 knockout mouse across 4 datasets.

Gene Symbol	Gene Name	logFC	AveExpr	t	P Value	adj.P.Val
Elf3	E74-like factor 3	-3.28	9.53	-29.35	7.48E-10	1.20E-06
Mgat4a	mannoside acetylglucosaminyltransferase 4, isoenzyme A	-2.21	9.93	-23.49	4.88E-09	3.70E-06
Mpzl2	myelin protein zero-like 2	-1.68	10.32	-22.70	6.51E-09	4.26E-06
Eps8l2	EPS8-like 2	-1.37	9.63	-20.14	1.77E-08	7.74E-06
Baiap2l1	BAI1-associated protein 2-like 1	-1.40	9.25	-17.86	4.81E-08	1.34E-05
Acsm3	acyl-CoA synthetase medium-chain family member 3	-1.91	7.43	-17.65	5.30E-08	1.36E-05
Pglyrp1	peptidoglycan recognition protein 1	-1.19	7.91	-16.28	1.04E-07	2.16E-05
Ckmt1	creatine kinase, mitochondrial 1, ubiquitous	-1.82	8.39	-16.24	1.06E-07	2.18E-05
Reep6	receptor accessory protein 6	-1.55	11.36	-15.60	1.47E-07	2.79E-05
Dgat2	diacylglycerol O-acyltransferase 2	-1.15	9.91	-15.31	1.72E-07	2.92E-05
BC025446	cDNA sequence BC025446	-1.56	7.07	-14.43	2.80E-07	3.88E-05
Grtp1	GH regulated TBC protein 1	-1.24	9.68	-14.21	3.18E-07	4.29E-05
Klf5	Kruppel-like factor 5	-1.32	11.02	-14.14	3.32E-07	4.43E-05
Lgals9	lectin, galactose binding, soluble 9	-1.52	9.91	-13.71	4.26E-07	5.02E-05
Sprr2a3	small proline-rich protein 2A3	-1.24	8.59	-13.64	4.44E-07	5.07E-05
Pllp	plasma membrane proteolipid	-1.67	7.79	-13.35	5.29E-07	5.52E-05
Tjp3	tight junction protein 3	-1.18	9.16	-13.28	5.55E-07	5.63E-05
Gprc5a	G protein-coupled receptor, family C, group 5, member A	-0.87	8.36	-12.83	7.33E-07	6.86E-05
Tspan8	tetraspanin 8	-1.57	8.97	-12.14	1.15E-06	9.82E-05
Mboat1	membrane bound O-acyltransferase domain containing 1	-1.13	10.06	-12.06	1.21E-06	9.99E-05
Sh2d4a	SH2 domain containing 4A	-1.34	8.19	-12.05	1.22E-06	9.99E-05
Smagp	small cell adhesion glycoprotein	-1.12	9.20	-12.07	1.21E-06	9.99E-05
Sprr2a1	small proline-rich protein 2A1	-1.68	9.26	-12.09	1.19E-06	9.99E-05

Tpd52	tumor protein D52	-0.89	10.25	-11.39	1.92E-06	1.37E-04
Lgals12	lectin, galactose binding, soluble 12	-0.98	7.26	-11.25	2.13E-06	1.46E-04
Krt7	keratin 7	-0.96	10.04	-11.04	2.47E-06	1.65E-04
2200002D01Rik	RIKEN cDNA 2200002D01 gene	-0.76	9.10	-10.78	2.97E-06	1.79E-04
St6galnac2		-0.95	10.18	-10.45	3.81E-06	2.14E-04
Pof1b	premature ovarian failure 1B	-1.37	6.51	-10.44	3.85E-06	2.15E-04
Rab17	RAB17, member RAS oncogene family	-1.32	9.19	-10.43	3.87E-06	2.15E-04
Cmbl	carboxymethylenebutenolidase-like (Pseudomonas)	-0.91	8.35	-10.40	3.98E-06	2.16E-04
Tspan1	tetraspanin 1	-1.22	7.50	-10.41	3.95E-06	2.16E-04
Ppl	periplakin	-0.74	8.70	-10.16	4.76E-06	2.45E-04
Krt8	keratin 8	-0.81	12.05	-9.98	5.50E-06	2.69E-04
Stard10	START domain containing 10	-0.79	11.92	-9.79	6.40E-06	2.98E-04
Myo5b	myosin VB	-1.41	9.89	-9.75	6.62E-06	3.02E-04
Lass3	LAG1 homolog, ceramide synthase 3	-1.46	7.49	-9.55	7.78E-06	3.39E-04
Grb7	growth factor receptor bound protein 7	-0.67	10.73	-9.39	8.86E-06	3.70E-04
Prr15l	proline rich 15-like	-0.84	8.82	-9.39	8.88E-06	3.70E-04
Fxyd3	FXYD domain-containing ion transport regulator 3	-0.98	8.58	-9.04	1.19E-05	4.71E-04
Ano9	anoctamin 9	-1.01	8.58	-8.79	1.49E-05	5.57E-04
Tacstd2	tumor-associated calcium signal transducer 2	-1.46	7.36	-8.68	1.65E-05	5.97E-04
Sh3bgrl2	SH3 domain binding glutamic acid-rich protein like 2	-0.59	9.73	-8.59	1.78E-05	6.27E-04
Gpx2	glutathione peroxidase 2	-2.32	8.59	-7.84	3.56E-05	1.05E-03
Trp53inp1	transformation related protein 53 inducible nuclear protein 1	0.58	10.46	7.84	3.59E-05	1.05E-03
Hmcn1	hemicentin 1	0.56	8.18	7.81	3.69E-05	1.06E-03
Ppargc1b	peroxisome proliferative activated receptor, gamma, coactivator 1 beta	-0.55	8.72	-7.80	3.71E-05	1.06E-03
Rarb	retinoic acid receptor, beta	0.67	10.83	7.67	4.20E-05	1.16E-03
Rassf7	Ras association (RalGDS/AF-6) domain family (N-terminal) member 7	-0.50	9.41	-7.61	4.48E-05	1.22E-03

Il17rc	interleukin 17 receptor C	-0.58	8.84	-7.46	5.20E-05	1.33E-03
Aim1l	absent in melanoma 1-like	-0.64	7.90	-7.32	5.95E-05	1.46E-03
AI661453	expressed sequence AI661453	-0.52	7.69	-7.24	6.52E-05	1.57E-03
Upk1b	uroplakin 1B	0.92	10.21	7.23	6.53E-05	1.57E-03
Nrn1	neuritin 1	-1.07	9.61	-7.19	6.85E-05	1.61E-03
Spry2	sprouty homolog 2 (Drosophila)	0.50	9.43	7.14	7.23E-05	1.67E-03
Rnpep	arginyl aminopeptidase (aminopeptidase B)	-0.50	11.50	-7.09	7.62E-05	1.74E-03
Hpse	heparanase	-0.63	8.14	-7.05	7.93E-05	1.79E-03
Fgfr3	fibroblast growth factor receptor 3	-0.48	8.78	-7.02	8.13E-05	1.81E-03
Aldh1a2	aldehyde dehydrogenase family 1, subfamily A2	0.54	12.15	6.96	8.69E-05	1.87E-03
Pparg	peroxisome proliferator activated receptor gamma	-1.20	9.53	-6.87	9.59E-05	2.01E-03
Dpysl4	dihydropyrimidinase-like 4	0.48	9.76	6.66	1.20E-04	2.35E-03
Wfdc2	WAP four-disulfide core domain 2	-0.56	9.48	-6.59	1.29E-04	2.48E-03
Cnksr2	connector enhancer of kinase suppressor of Ras 2	0.53	8.63	6.53	1.39E-04	2.62E-03
Elovl1	elongation of very long chain fatty acids (FEN1/Elo2, SUR4/Elo3, yeast)-like 1	-0.44	11.51	-6.48	1.47E-04	2.70E-03
Pwwp2b	PWWP domain containing 2B	-0.44	8.60	-6.46	1.50E-04	2.75E-03
Msln	mesothelin	-0.53	7.68	-6.45	1.51E-04	2.76E-03
Sept3	septin 3	0.52	10.67	6.45	1.52E-04	2.77E-03
Serinc2	serine incorporator 2	0.67	10.74	6.30	1.78E-04	3.12E-03
Prr13	proline rich 13	-0.51	9.81	-6.25	1.90E-04	3.28E-03
Jakmip1	janus kinase and microtubule interacting protein 1	-0.52	8.30	-6.17	2.07E-04	3.49E-03
Ezr	ezrin	-0.40	11.57	-6.00	2.54E-04	4.03E-03
Slc25a10	solute carrier family 25 (mitochondrial carrier, dicarboxylate transporter), member 10	-0.40	10.43	-5.98	2.59E-04	4.09E-03
Tinagl1	tubulointerstitial nephritis antigen-like 1	-0.57	10.02	-5.96	2.65E-04	4.15E-03
Perp	PERP, TP53 apoptosis effector	-0.86	8.38	-5.93	2.76E-04	4.27E-03
Cdkn1c	cyclin-dependent kinase inhibitor 1C (P57)	0.54	11.74	5.91	2.80E-04	4.30E-03

Atrnl1	attractin like 1	0.48	10.85	5.82	3.12E-04	4.66E-03
St8sia1	ST8 alpha-N-acetyl-neuraminide alpha-2,8-sialyltransferase 1	0.74	9.15	5.73	3.49E-04	5.05E-03
Sulf2	sulfatase 2	0.29	12.49	3.91	3.95E-03	0.03
S100a14	S100 calcium binding protein A14	-0.29	6.07	-2.69	0.03	0.08
Gsn	gelsolin	0.19	10.77	2.60	0.03	0.09
Slc28a3	solute carrier family 28 (sodium-coupled nucleoside transporter), member 3	0.17	6.38	1.77	0.11	0.23
Tmprss2	transmembrane protease, serine 2	-0.07	10.00	-0.71	0.49	0.65
Scd1	stearoyl-Coenzyme A desaturase 1	0.09	10.18	0.70	0.50	0.66
Hsd17b14	hydroxysteroid (17-beta) dehydrogenase 14	Unavailable	Unavailable	Unavailable	Unavailable	Unavailable

*NOTE: All statistics are from dataset GSE39624.

Supplement figure

Supplement figure. The relation between KLF5 and cell proliferation.

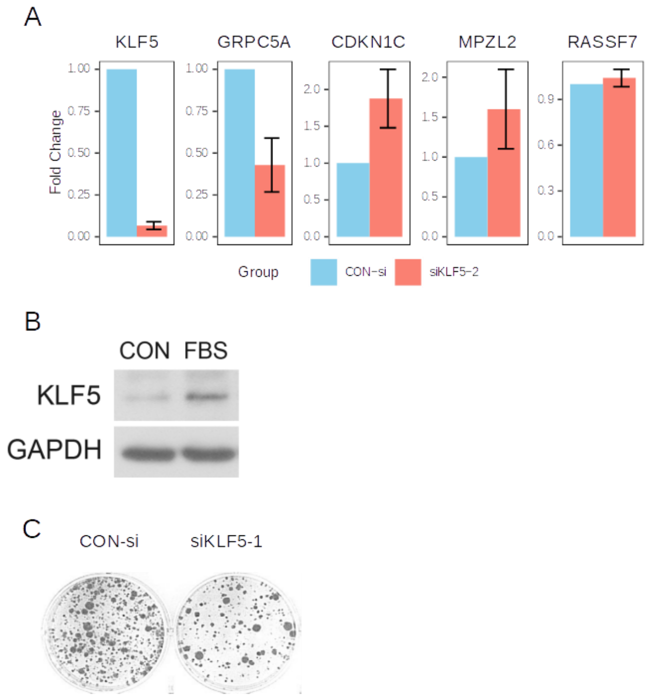

Supplement figure. The relation between KLF5 and cell proliferation. A, KLF5 silencing by siKLF5-2 and its effect on the expression of four genes. B, starvation assay showed that the expression of KLF5 increased

significantly upon FBS induction. C, clone formation assay showed SGC7901 formed much fewer clones after treatment with KLF5 siRNA.

Supplement references

1. Azami, T.; Waku, T.; Matsumoto, K.; Jeon, H.; Muratani, M.; Kawashima, A.; Yanagisawa, J.; Manabe, I.; Nagai, R.; Kunath, T.; et al. Klf5 maintains the balance of primitive endoderm versus epiblast specification during mouse embryonic development by suppression of Fgf4. Development 2017, 144, 3706–3718, doi:10.1242/dev.150755.

2. Xing, C.; Ci, X.; Sun, X.; Fu, X.; Zhang, Z.; Dong, E.N.; Hao, Z.-Z.; Dong, J.-T. Klf5 deletion promotes Pten deletion-initiated luminal-type mouse prostate tumors through multiple oncogenic signaling pathways. Neoplasia 2014, 16, 883–99, doi:10.1016/j.neo.2014.09.006.

3. Bell, S.M.; Zhang, L.; Mendell, A.; Xu, Y.; Haitchi, H.M.; Lessard, J.L.; Whitsett, J.A. Kruppel-like factor 5 is required for formation and differentiation of the bladder urothelium. Dev. Biol. 2011, 358, 79–90, doi:10.1016/j.ydbio.2011.07.020.
